# Differences between the clinical and histopathological tumor stages in breast cancer diagnosed using vacuum-assisted breast biopsy

**DOI:** 10.3892/ol.2015.2945

**Published:** 2015-02-09

**Authors:** HAI-LIN PARK, JI-SUN HONG, SO YONG CHANG, JUNG YIN HUH, JI EUN SHIN, JI-YOUNG KIM, JEONG YUN SHIM, SONGMI NOH

**Affiliations:** 1 Department of Surgery, Kangnam CHA Hospital, CHA University College of Medicine, Seoul 135-080, Republic of Korea; 2 Department of Diagnostic Radiology, Kangnam CHA Hospital, CHA University College of Medicine, Seoul 135-080, Republic of Korea; 3 Department of Pathology, Kangnam CHA Hospital, CHA University College of Medicine, Seoul 135-080, Republic of Korea

**Keywords:** breast cancer, vacuum-assisted breast biopsy, T stage

## Abstract

The present study aimed to determine the difference between the clinical tumor stage (T stage) based on pre-operative ultrasound and the histopathological T stage subsequent to surgery in vacuum-assisted breast biopsy (VABB)-diagnosed breast cancer. Tumor sizes measured ultrasonography (USG) and histologically were retrospectively calculated and analyzed using paired t-tests in 209 patients diagnosed with breast cancer using VABB. The patients were classified into two groups, consisting of breast imaging reporting and data system (BI-RADS) category 4a or below, who underwent complete resection by VABB, and BI-RADS category 4b or above, who underwent incisional biopsy by VABB. The histopathological tumor size was found to be smaller compared with the USG-determined size in 92.3% of pT1a, 75.5% of pT1b, 44.2% of pT1c, 47.7% of pT2 and 0% of pT3 cases. Furthermore, the histopathological tumor size was smaller compared with the USG-determined size in 62.8% of cases classified as BI-RADS category 3–4a and in 53.7% of cases classified as BI-RADS category 4b-5. The smaller the primary tumor at the time of diagnosis by VABB, the higher the likelihood of pathological underestimation on post-operative histopathological assessment compared to pre-operative USG.

## Introduction

The clinical tumor size (cT), which is determined by palpation during physical examination or by imaging, is important in determining patient prognosis and treatment (1–4). Clinical staging determines the necessity of pre-operative chemotherapy and sentinel lymph node biopsy. However, the methods used to determine tumor size can yield various results, which in turn can affect treatment options and ultimately, patient outcomes. For example, clinical staging based on physical examination is subjective due to the examiner. This method is also not effective for nonpalpable lesions (5). Breast imaging methods, which include mammography, ultrasonography and magnetic resonance imaging (MRI), are less subjective, but also demonstrate certain limitations. The tumor boundary in mammography is often unclear in dense breast tissues, particularly in Asian or young women, and the tumor size may vary depending on the type of mammography performed. Additionally, numerous studies indicate that mammography underestimates the true tumor size compared to the histopathological size (6–8). By contrast, breast ultrasonography allows for easier measurement of the longest tumor dimension and is strongly correlated with the histopathological size compared to physical examination or mammography, although numerous studies indicate that ultrasonography (USG) also underestimates the tumor size compared with the histopathological size (6,7).

In contrast to cT, the pathological tumor size (pT) is determined by microscopic measurement of surgical specimens, and accurate pathological tumor staging (T staging) plays a decisive role in determining whether to perform post-operative adjuvant chemotherapy. Among biopsy methods, vacuum-assisted breast biopsy (VABB), a more recently developed form of core needle biopsy, is now widely used. This biopsy allows complete excision of target lesions, with accuracy similar to that of excisional biopsy. However, the fragmented specimens create certain challenges in histopathological assessment (9). Based on the importance of obtaining reliable information regarding tumor size to accurately determine cancer stage, the purpose of the present study was to investigate any differences between pre-operative clinical T stage and histopathological T stage subsequent to surgery in VABB-diagnosed breast cancer.

## Materials and methods

The present retrospective study was conducted using the medical records of 209 patients out of 294 potential participants diagnosed with invasive breast cancer by VABB (Mammotome^®^; Devicor Medical Products, Inc., Cincinnati, OH, USA) at the Department of Surgery of Kangnam CHA Hospital, CHA University College of Medicine (Seoul, Republic of Korea) between January 2007 and December 2012. The remaining 85 patients were excluded due to the cases reporting the use of neoadjuvant chemotherapy, or the presence of ductal carcinoma *in situ* or invasive breast cancer with extensive intraductal components. All patients underwent surgical resection of breast cancer, comprising modified radical mastectomy or breast-conserving surgery, with or without sentinel lymph node biopsy. VABB was performed using an eight-gauge needle on USG-assessed lesions classified as breast imaging reporting and data system (BI-RADS) categories 3, 4a-c and 5. Complete excisional biopsy was performed for USG-assessed lesions categorized as category 3 or 4a, but only incisional biopsy using VABB was performed to obtain 3–5 core tissue samples in lesions that were classified as category 4b and above. The histological tumor size was measured in the long axis. The presence of synchronous cancers and the extent of retroareolar involvement were recorded using sonograms.

Breast ultrasonography was performed using duplex sonography that uses B-mode and color Doppler with a probe of 7–12 MHz high frequency Linear Array (Logic 700; GE Healthcare Bio-sciences, Pittsburgh, PA, USA; HDI 5000; Philips Ultrasound, Bothell, WA, USA). The tumor size was determined based on the longest dimension of the measured tumor, although the long cytoplasmic processes of the tumor were excluded from measurement. Sagittal and transverse views were obtained for each mass, and the longest dimension was obtained for each transducer position. Whenever possible, the longest dimension was obtained collinear to the ultrasound beam. The present study was performed with the approval of the Institutional Ethical Committee of Kangnam CHA University Hospital (approval number, KNC13-016).

### Statistical analysis

All statistical analyses were performed using SPSS version 14.0 (SPSS Inc., Chicago, IL, USA), and P<0.05 was considered to indicate a statistically significant difference. The tumor sizes that were measured using USG and histology were calculated and analyzed by paired t-tests. Bivariate simple correlation analysis of the tumor size was performed using Pearson’s correlation coefficient. Pearson’s correlation coefficient was analyzed between the tumor size in the final pathological result subsequent to surgery and the pre-operative tumor size on USG.

## Results

In total, 209 patients were enrolled in the present study. These patients were classified by the ultrasound BI-RADS categorization as shown in Table I. A comparison in size between the clinical T staging performed using USG and the final pathological T stage subsequent to surgery revealed that the pathological tumor size was smaller than the USG-determined size in the majority of cases (114 out of 209 patients; 54.5%). The pathological tumor size and USG-determined size were equal in 34 cases (16.3%), while the pathological tumor size was larger than the USG-determined size in 61 cases (29.2%) (Table II). Further analysis of these results by T staging revealed that the pathological tumor size was smaller than the USG-determined size in 12 out of 13 pT1a cases (92.3%). In addition, the pathological tumor size was smaller than the USG-determined size in 37 of the 49 pT1b cases (75.5%). This was also reported in 34 out of 77 pT1c cases (44.2%) and in 31 out of 65 pT2 cases (47.7%), but was not observed in the three pT3 cases (0.0%).

Taken together, these findings indicate that the larger the size of the primary tumor, the lower the possibility of histological underestimation. This is likely due to the volume of specimens excised by VABB being relatively small, while the residual lesion of larger primary tumors exists in a wide range, making pathological measurement easier.

**Figure 1:**
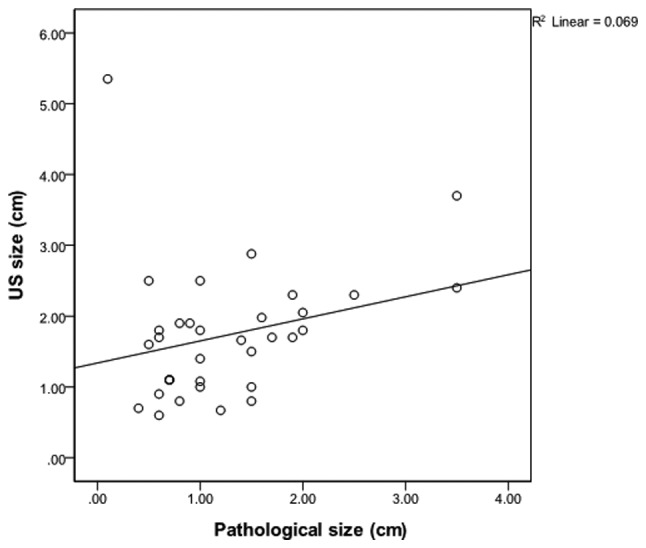
Bivariate correlation analysis of post-operative final pathological tumor size and initial ultrasonography-determined size in US category 3–4a lesions [correlation coefficient (r)=0.262; P=0.129, r^2^=0.069]. US, ultrasound.

**Figure 2:**
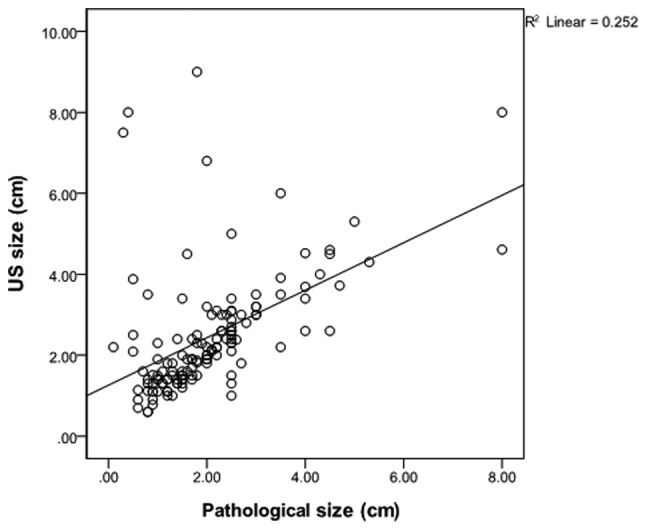
Bivariate correlation analysis of the post-operative final pathological size and the initial ultrasonography-determined size in US category 4b-5 lesions [correlation coefficient (r)=0.502; P<0.01; r^2^=0.252]. US, ultrasound.

Analysis on ultrasound BI-RADS categorization revealed that in 27 of 43 category 3–4a cases (62.8%), in which complete excision by VABB, the pathological tumor size was smaller than the USG-determined size, while only 10 cases (23.3%) revealed the opposite result (Table III). An analysis of the aforementioned results by T staging also demonstrated that the pathological tumor size was smaller than the USG-determined size in 100% of pT1a cases, 77.8% of pT1b cases, 33.3% of pT1c cases, 66.7% of pT2 cases and 0% of pT3 cases, again indicating that the bigger the pathological tumor size, the less likely it is that histological underestimation occurs. However, 88 out of 164 cases (53.7%) in category 4b-5, where an incisional biopsy by VABB was performed, revealed that the pathological tumor size was smaller than the USG-determined size (Table IV). Further analysis of the category 4b-5 results by tumor-node-metastasis (TNM) staging showed that the pathological tumor size was smaller than the USG-determined size in 88.9% of pT1a cases, 82.8% of pT1b cases, 46.8% of pT1c cases, 45.8% of pT2 cases and 0.0% of pT3 cases, confirming that the larger the pathological tumor size, the less likely it is that histological underestimation takes place. Simple correlation analysis on the category 3–4a and 4b-5 groups revealed that the correlation coefficient of the category 3–4a group was 0.262 (P=0.129), which was lower than the coefficient of 0.502 (P<0.01) identified in the category 4b-5 group (Figs. 1 and 2). These findings indicate that histological underestimation occurs more commonly when a target lesion is confirmed as malignant following complete excision of the USG category 3 or 4a lesion using VABB compared with incisional biopsy only for lesions in USG category 4b or above.

## Discussion

Tumor size is essential not only for determining the clinical stage prior to surgery, assessing the requirement for pre-operative chemotherapy and deciding whether to perform sentinel lymph node biopsy, but is also crucial for determining the stage of tumors, the prognosis for the patient and the necessary post-operative adjuvant therapy (4,10,11). Tumor size can be measured by physical examination, mammography, USG and MRI prior to surgery and by pathological T staging subsequent to surgery. Physical examination is an easy, simple and economical measurement method that yields immediate results. However, it is limited in cases involving nonpalpable breast masses that are clinically latent deep within the breast tissue (5), and the assessment can be subjective based on factors such as the examiner and obesity (12). Mammography is a more objective measurement for even nonpalpable breast cancer and it is also less affected by either the patient or the examiner (6). However, the determined tumor size can be inconsistent depending on the distance between the breast tumor and the X-ray film, and the maximal diameter of the tumor can be inaccurate in the plane dimension (7). Accurate measurement is also limited in young and premenopausal women with dense breast tissues, or in cases of tumors with long and slender cytoplasmic processes and unclear boundaries (6–8). By contrast, breast sonography is known to be more useful in patients with dense breast tissues (8), and its usage has been increasing. Breast USG allows for the measurement of the longest dimension of a breast mass from various directions, with no artificially enlarged image of a mass and no radiation exposure. However, breast USG is a relatively subjective procedure that depends on the examiner, resulting in non-reproducible results. In addition, the boundary of the mass must be clear to ensure accurate measurement (6,7).

Despite these limitations, USG measurements of the mass size are usually more accurate than physical examination or mammography (13–15). Forouhi *et al* reported that the USG-determined size demonstrated an improved correlation with the histological size (correlation coefficient, 0.89) compared to the size obtained by physical examination or mammography (9). Other studies have also reported that breast ultrasonography can measure the mass size with the greatest accuracy, demonstrating correlation coefficients with physical examination or mammography of 0.84 and 0.80, respectively (6,16). Choi *et al* demonstrated that the correlation coefficient with the histological size was 0.83, which is significantly higher compared with the value obtained by physical examination or mammography, demonstrating that breast USG is the most accurate measurement of breast masses (7). With the increased used of VABB for the diagnosis of breast cancer, measurement of the histopathological size has become increasingly challenging. The present study aimed to identify the impact of VABB on tumor size measurements by comparing the pre-operative USG-determined size with the post-operative histopathological size in 209 patients with invasive breast cancer who underwent surgery subsequent to tissue biopsy by VABB.

The USG-determined breast mass size strongly correlates with the histological tumor size, although it tends to underestimate the pathological size (7,17–19). Several studies have reported that mass size has been underestimated in ~80% of cases by breast USG (6,9,16). Breast USG particularly underestimates tumors 2 cm in size. In addition, Lee *et al* reported that physical examination, mammography and USG tended to underestimate tumor size (20). This was attributed to the fact that patients involved in the study possessed relatively large tumors that were all clinically palpable.

In the present study, the USG-determined and pathological tumor sizes were compared, revealing 148 cases (70.8%) in which the tumor sizes were equal in size between USG and pathological analysis or overestimated by USG, while numerous other studies have revealed a tendency of underestimation in USG. This is due to the measured pathological size being smaller than the primary lesion, since the pathological size represents the measurement of the residual lesion subsequent to biopsy using VABB. In comparison with the USG-determined size, the pathological size was found to be smaller in 114 cases (54.5%) in the present study. The pathological tumor size was smaller than the USG-determined size in 92.3% of pT1a cases, 75.5% of pT1b cases, 44.2% of pT1c cases and 47.7% of pT2 cases, indicating that the measurement of tumor size is smaller pathologically compared with USG when the primary tumor appears smaller clinically. By contrast, the histopathological tumor size was larger than the USG-determined size in 61 cases (29.2%) and equivalent in 34 cases (16.3%). This may be due to a large portion of the small primary lesion being removed by VABB and it may have been challenging to exactly measure the pathological T size with the fragmented specimens.

The VABB procedure varies depending on the USG BI-RADS category. Usually complete excision biopsy is performed in category 3–4a cases. However, incisional biopsy is performed to obtain 3–5 core tissue samples in category 4b-5 cases. As a result, the present study divided the cases into two groups by procedure, category 3–4a and category 4b-5. The USG measurement and the pathological measurement were compared in each group. In category 3 and 4a, 27 cases (62.8%) exhibited a pathological size that was smaller than the USG-determined size. The pathological size measurement was smaller than the USG-determined size in 100% of pT1a, 77.8% of pTlb, 33.3% of pT1c and 66.7% of pT2 cases. In category 4b-5, 88 cases (53.7%) had a pathological size that was smaller than the USG-determined size. The percentage of cases was 88.9% in the pT1a, 82.8% in the pTlb, 46.8% in the pT1c and 45.8% in the pT2 categories. In these two groups, the pathological size was also smaller than the USG-determined size when the size of the primary lesion was small. Simple correlational analysis of the pathological and USG-determined sizes revealed that the correlation coefficient in categories 3–4a was 0.262 (P=0.129), which was not significant, although a weak-positive linear correlation was observed. By contrast, the correlation coefficient in category 4b-5 was 0.502 (P<0.01), which demonstrated a clear positive linear correlation.

The square of the correlation coefficient is described in terms of explanatory power, which refers to the variable importance or impact of each variable. Using this, dependence between the pathological size and the USG-determined size can be demonstrated. The explanatory power of categories 3–4a and categories 4b-5 was 6% and 25%, respectively. In categories 3–4a, where a complete excision was performed, the dependence between the pathological size and the USG-determined size is low due to the pathological underestimation. In the case of a large tumor, the residual lesion did not demonstrate a large difference, as the amount of the lesion removed by VABB is not relatively large. However, in the case of a small tumor, a possibility remains that the size of the residual lesion can be measured as smaller than the original size. In these cases, histopathological T staging is underestimated, possibly influencing whether to implement adjuvant therapy in the future. Taken together, these data demonstrate that the smaller the primary tumor in lesions classified as category 3–4a, the higher the likelihood of pathological underestimation between pre-operative USG and post-operative histopathological tumor sizes. This underestimation can lead to omission of necessary adjuvant chemotherapy and underlines the importance of considering the size of clinical lesions properly when staging tumors.

## Figures and Tables

**Table I:** Breast imaging reporting and data system usltrasonography categories of invasive breast cancer diagnosed by vacuum-assisted breast biopsy.

Category	Patients, n (%)
3	7 (3.3)
4a	36 (17.2)
4b	44 (21.1)
4c	47 (22.5)
5	75 (35.9)
Total	209 (100)

**Table II:** Overall comparison between the post-operative permanent pathological size and the initial USG-determined size.

		Pathological size vs. USG-determined size, n (%)			
			
T Stage	Total, n	Larger	Equal	Smaller	P-value
pT1a	13	0 (0.0)	1 (7.7)	12 (92.3)	0.003
pT1b	49	4 (8.2)	8 (16.3)	37 (75.5)	0.0001
pT1c	77	29 (37.7)	14 (18.2)	34 (44.2)	0.0161
pT2	65	24 (36.9)	10 (15.4)	31 (47.7)	0.9337
pT3	5	4 (80.0)	1 (20.0)	0 (0.0)	0.2829
Total	209	61 (29.2)	34 (16.3)	114 (54.5)	0.0001

T, tumor; USG, ultrasonography.

**Table III:** Comparison between the post-operative permanent pathological size and initial USG-determined size in USG category 3–4a lesions.

		Pathological size vs. USG-determined size, n (%)			
			
T Stage	Total, n	Larger	Equal	Smaller	P-value
pT1a	4	0 (0.0)	0 (0.0)	4 (100.00)	0.141
pT1b	18	0 (0.0)	4 (22.2)	14 (77.8)	0.0001
pT1c	15	8 (53.3)	2 (13.3)	5 (33.3)	0.862
pT2	6	2 (33.3)	0 (0.0)	4 (66.7)	0.441
pT3	0	0 (0.0)	0 (0.0)	0 (0.0)	
Total	43	10 (23.3)	6 (14.0)	27 (62.8)	0.01

T, tumor; USG, ultrasonography.

**Table IV:** Comparison between the post-operative permanent pathological size and the initial USG-determined size in USG category 4b-5 lesions.

		Pathological size vs. USG-determined size, n (%)			
			
T Stage	Total, n	Larger	Equal	Smaller	P-value
pT1a	9	0 (0.0)	1 (11.1)	8 (88.9)	0.016
pT1b	29	1 (3.4)	4 (13.8)	24 (82.8)	0.002
pT1c	62	21 (33.9)	12 (19.3)	29 (46.8)	0.015
pT2	52	22 (37.3)	10 (16.9)	27 (45.8)	0.783
pT3	5	4 (80.0)	1 (20.0)	0 (0.0)	0.283
Total	164	48 (29.3)	28 (17.1)	88 (53.7)	0.001

T, tumor; USG, ultrasonography.

## References

[1] Veronesi U, Galimberti V, Zurrida S, Pigatto F (2001). Sentinel lymph node biopsy as an indicator for axillary dissection in early breast cancer. Eur J Cancer.

[2] Cowen D, Jacquemier J, Houvenaeghel G (1998). Local and distant recurrence after conservative management of ‘very low-risk’ breast cancer are dependent events: a 10-year follow-up. Int J Radiat Oncol Biol Phys.

[3] van Dongen JA, Bartelink H, Fentiman IS (1992). Factors influencing local relapse and survival and results of salvage treatment after breast-conserving therapy in operable breast cancer: EORTC trial 10801, breast conservation compared with mastectomy in TNM stage I and II breast cancer. Eur J Cancer.

[4] Carter CL, Allen C, Henson DE (1989). Relation of tumor size, lymph node status, and survival in 24,740 breast cancer cases. Cancer.

[5] Hieken TJ, Harrison J, Herreros J, Velasco JM (2001). Correlating sonography, mammography, and pathology in the assessment of breast cancer size. Am J Surg.

[6] Fornage BD, Toubas O, Morel M (1987). Clinical, mammographic, and sonographic determination of preoperative breast cancer size. Cancer.

[7] Choi KH, Bae JW, Lee JB, Koo BH (1999). Clinical, mammographic, and ultrasonographic assessment of breast cancer sizes. J Korean Breast Cancer Soc.

[8] Fei SA (1992). Breast masses. Mammographic and sonographic evaluation. Radiol Clin North Am.

[9] Forouhi P, Walsh JS, Anderson TJ, Chetty U (1994). Ultrasonography as a method of measuring breast tumour size and monitoring response to primary systemic treatment. Br J Surg.

[10] Clark GM (1992). Integrating prognostic factors. Breast Cancer Res Treat.

[11] Maehle BO, Skjaerven R (1984). Prediction of prognosis in axillary lymph node positive breast cancer patients: a statistical study. Br J Surg.

[12] Dixon JM, Senbanjo RO, Anderson TJ, Forrest AP, Elton RA (1984). Clinical assessment of tumour size in primary breast carcinoma. Clin Oncol.

[13] Lambie RW, Hodgden D, Herman EM, Kopperman M (1983). Sonomammographic detection of lobular carcinoma not demonstrated on xeromammography. J Clin Ultrasound.

[14] Warwick DJ, Smallwood JA, Guyer PB, Dewbury KC, Taylor I (1988). Ultrasound mammography in the management of breast cancer. Br J Surg.

[15] Nishimura S, Matsusue S, Koizumi S, Kashihara S (1988). Size of breast cancer on ultrasonography, cut-surface of resected specimen, and palpation. Ultrasound Med Bio.

[16] Pierie JP, Perre CI, Levert LM, de Hooge P (1998). Clinical assessment, mammography and ultrasonography as methods of measuring the size of breast cancer: a comparison. The Breast.

[17] Hwang KT, Kim HY, Chung JK (2010). A comparative study between the preoperative diagnostic tumor size and the postoperative pathologic tumor size in patients with breast tumors. J Breast Cancer.

[18] Bosch AM, Kessels AG, Beets GL, Rupa JD, Koster D, van Engelshoven JM, von Meyenfeldt MF (2003). Preoperative estimation of the pathological breast tumour size by physical examination, mammography and ultrasound: a prospective study on 105 invasive tumours. Eur J Radiol.

[19] Meden H, Neues KP, Röben-Kämpken S, Kuhn W (1995). A clinical, mammographic, sonographic and histologic evaluation of breast cancer. Int J Gynaecol Obstet.

[20] Lee CS, Bong JG, Park JH (2003). The accuracy of the physical examination, mammography, and ultrasonography in the assessment of tumor size and axillary lymph node metastasis in breast cancer patients. J Korean Breast Cancer Soc.

